# Current Knowledge and Future Challenges in Takotsubo Syndrome: Part 2—Treatment and Prognosis

**DOI:** 10.3390/jcm10030468

**Published:** 2021-01-26

**Authors:** Francesco Santoro, Adriana Mallardi, Alessandra Leopizzi, Enrica Vitale, Elias Rawish, Thomas Stiermaier, Ingo Eitel, Natale D. Brunetti

**Affiliations:** 1 Department of Medical and Surgical Sciences, University of Foggia, 71122 Foggia, Italy; adriana.mallardi3@gmail.com (A.M.); Alessandraleopizzi91@gmail.com (A.L.); enri91@gmail.com (E.V.); natale.brunetti@unifg.it (N.D.B.); 2 Medical Clinic II (Cardiology/Angiology/Intensive Care Medicine), University Heart Center, 23552 Lübeck, Germany; elias.rawish@uksh.de (E.R.) thomas.stiermaier@uksh.de (T.S.); ingo.eitel@uksh.de (I.E.); 3 German Center for Cardiovascular Research (DZHK), 23552 Lübeck, Germany

**Keywords:** takotsubo syndrome, apical ballooning, therapy, prognosis, outcome

## Abstract

Takotsubo syndrome (TTS) represents a form of acute heart failure featured by reversible left ventricular systolic dysfunction. The management during the acute phase is mainly performed with supportive pharmacological (diuretics, ACE-inhibitors/angiotensin-receptor blockers (ARBs), anticoagulants, antiarrhythmics, non-catecholamine inotropics (levosimendan), and non-pharmacological (mechanical circulatory and respiratory support) therapy, due to the wide clinical presentation and course of the disease. However, there is a gap in evidence and there are no randomized and adequately powered studies on clinical effectiveness of therapeutic approaches. Some evidence supports the use ACE-inhibitors/ARBs at long-term. A tailored approach based on cardiovascular and non-cardiovascular risk factors is strongly suggested for long-term management. The urgent need for evidence-based treatment approaches is also reflected by the prognosis following TTS. The acute phase of the disease can be accompanied by various cardiovascular complications. In addition, long term outcome of TTS patients is also related to non-cardiovascular comorbidities. Physical triggers such as hypoxia and acute neurological disorders in TTS are associated with a poor outcome.

## 1. Acute Treatment in Takotsubo Syndrome (TTS)

Although Takotsubo syndrome (TTS) has been classically described as a benign condition, life-threatening acute complications have been described in 52% of patients, while 11% have more than two complications that contribute to higher mortality rate [1,2,3]. In-hospital death rate ranges from 2.4 to 4.1%, while rates of pulmonary edema and cardiogenic shock range from 6 to 9% and 9 to 11.4%, respectively [1,3,4].

In the acute phase, patients are generally treated with several supportive measures, pharmacological (beta-blockers, diuretics, anticoagulants, antiarrhythmics, non-catecholamine inotropics (levosimendan)), and nonpharmacological (mechanical circulatory and respiratory support), depending on the grade of heart failure and the existence of concomitant complications.

According to current literature “mild” form of TTS without the existence of acute heart failure require only therapy for comorbidities. However, no dedicated randomized trial has been published so far and few data come from adequately powered studies [5].

Beta-blockers were commonly used in the acute phase of TTS due to the possibility of blocking adrenergic surge effects. Actually, no benefit for beta-blockers at short- and long-term has been proven in TTS [5,6]. In a retrospective nationwide study, Isogai et al. found no significant association between early beta-blockers use (within first 2 days after admission) and in-hospital mortality in patients with TTS; there is therefore no evidence to support early beta-blockers use as routine practice in acute-phase management of TTS [7]. Short acting beta 1 blockers can be used only in case of left ventricular outflow-tract obstruction (LVOTO). Experimental data showed high density of β2-adrenergic receptors in the apical and midventricular segments of the left ventricle, while higher concentrations of β1-adrenergic receptors can be found in basal LV segments [8].

Esmolol, a β-1 cardio-selective blocker with a short half-life (9 min), may be an easily manageable tool for the treatment of patients with TTS and LVOTO. Santoro et al. found in a case series of 10 patients with TTS and LVOTO that Esmolol infusion was associated with a reduction of the intraventricular gradient (before infusion 47 ± 16 mmHg and after 18 ± 2 mm Hg, *p* = 0.01) and systemic blood pressure [9]. No adverse events were observed during esmolol infusion.

### 1.1. Treatment of Acute Heart Failure in TTS

The reported incidence of cardiogenic shock (CS) in TTS ranges from 6% to 20% [10,11,12]. Acute management of CS in TTS is challenging because usual therapeutic options (e.g., catecholamines which are routinely advocated in cases of circulatory compromise) can be potentially harmful [13]. TTS patients treated with i.v. catecholamine may show highly compromised circulation and cardiac function, poorer in-hospital outcomes, and higher long-term mortality rates in comparison to patients not receiving any form of catecholamine support [14]. Again, it has to be taken into account that these data are derived from non-randomized studies.

However, considering the pathophysiology of the syndrome, the Heart Failure Association of the European Society of Cardiology (ESC) has recommended to avoid or withdraw exogenous catecholamines as they could probably prolong or exacerbate the acute phase of the syndrome by activating catecholamine receptors or their downstream molecular pathways [15].

Levosimendan is a non-catecholaminergic inotropic drug, commonly used in patients with acute heart failure; the drug may represent an option for TTS patients, but its use, is limited to patients with impaired systolic function, without LVOTO and with systolic arterial pressure ≥ 90 mm Hg. Clinical data from case series showed the safety and feasibility of this treatment in TTS patients [16]; no adverse events were reported, LVEF improved in all patients and none developed LVOTO. Levosimendan infusion should be performed preferably for 24 h at an infusion rate of 0.1 mg/kg/min without loading dose. Invasive hemodynamic and ECG monitoring should be performed during infusion and for the following 24 h due to a minimal risk of hypotension [17]. Moreover, although not reportedly in TTS patients, levosimendan infusion may be associated with an increased risk of tachy-arrhythmias [18].

Mechanical circulatory support (MCS) should be evaluated early in patients with TTS and cardiogenic shock. Intra-aortic balloon pump (IABP), TandemHeart, extracorporeal membrane oxygenation (ECMO), and microaxial pumps (i.e., Impella™, Abiomed) are used in a bridge-to-recovery strategy [19].

IABP use has been evaluated in a European registry of 2250 TTS patients, where 42 patients with CS were treated with this approach. IABP use did not provide any benefit in term of in-hospital mortality, length of hospitalization, and need of invasive ventilation [20].

Recent data showed that there is a growing use of V-A ECMO and Impella in TTS patients with CS [21]. However, prospective studies are needed to evaluate the efficacy of different devices as well as their timing in this population.

**Figure 1:**
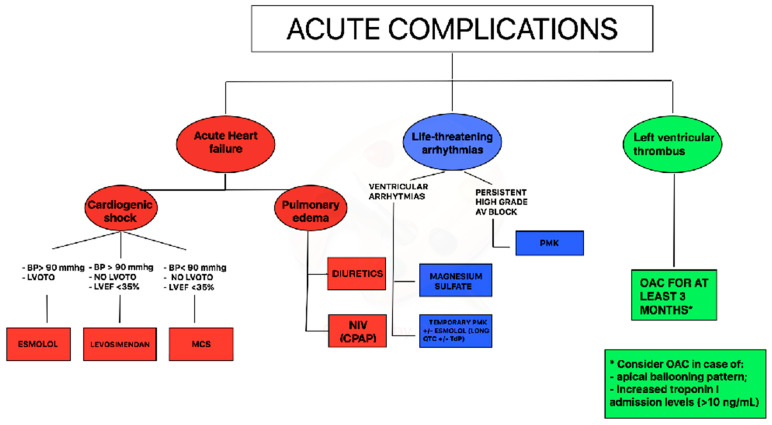
Proposal of acute complications management during Takotsubo syndrome (TTS) syndrome. Legend: AV = atrioventricular, BP = blood pressure, CPAP = Continuous positive airway pressure, LVOTO = left ventricular outflow tract obstruction, LVEF = left ventricular ejection fraction, MCS = mechanical circulatory support, NIV = non-invasive ventilation OAC = oral anticoagulation, PMK = pacemaker.

In TTS with impaired EF and cardiogenic shock a practical diagnostic and therapeutic flow chart can be suggested. In case of systolic blood pressure >90 mm Hg and left ventricular outflow tract obstruction, infusion of β1-selective beta-blockers (preferably with short half-life) could be considered; if systolic blood pressure is > 90 mmHg in the absence of left ventricular outflow tract obstruction, levosimendan infusion may be considered; finally, in case of cardiogenic shock with blood pressure < 90 mmHg, without left ventricular outflow tract obstruction mechanical circulating support could be considered (Figure 1).

### 1.2. Treatment of Arrhythmias in TTS

Life-threatening arrhythmias may occur during the acute phase of TTS in up to 8% of cases [22]. Data from the international multicenter GEIST registry suggest that the most common adverse rhythm disorders are ventricular arrhythmias and complete atrio-ventricular (AV) block [23], meanwhile InterTak registry showed an incidence of cardiac arrest in hospitalized patients of 8.1% [24].

Acute management of ventricular tachycardia includes a pharmacological approach with magnesium sulfate and/or short-acting β1-blocker or may require direct current cardioversion in case of sustained pulseless ventricular tachycardia. Of note, due to the high prevalence of prolonged QT interval in TTS patients during the acute phase [25], the use of amiodarone or sotalol needs to be evaluated for each case, after ECG recording. Moreover, all drugs potentially prolonging the QT interval such as antidepressants or antibiotics should be withdrawn immediately. In presence of torsade de pointes associated with QT-prolongation during hospitalization, short-acting β1-blocker (esmolol) or temporary pacing (in case of coexistent bradycardia) could represent an option (Figure 1).

Persistent high-grade AV block should be managed with permanent pacemaker implantation. All TTS patients with complete AV block who received a pacemaker showed a permanent pacing over follow-up suggesting that TTS probably occurred secondarily due to the rhythm disorder [26]; patients implanted with a permanent ICD, instead, did not receive any therapy at follow-up for malignant arrhythmias [27]. A careful assessment before ICD implantation is due in TTS patients, even in the case of severely reduced LVEF and concomitant ventricular tachyarrhythmias at index event. In the acute phase of TTS with enhanced vulnerability for adverse rhythm disorders, wearable defibrillator wests might also represent an option to prevent adverse heart rhythm events without permanent devices.

### 1.3. Treatment of Thromboembolic Events in TTS

LV thrombus formation has been reported in about 2.5% of all TTS patients and cardioembolic complications occur in 0.8% of this population [28]. Time of LV thrombi formation ranges from admission until 2 weeks after. Apical ballooning pattern, left ventricular ejection fraction ≤ 30%, previous vascular disease, increased C-protein levels, troponin-I levels > 10 ng/mL, and a white blood cell count on admission > 10 × 10^3^ cells/μL are independent predictors of LV thrombosis [28,29,30].

A simplified approach regarding OAC therapy in patients with TTS through echocardiographic evaluation combined with laboratory data (troponin levels) should be used for the acute management (Figure 1). In case of an apical ballooning pattern and increased troponin-I admission levels (> 10 ng/mL), prophylactic OAC could be considered, also on the possibility of late thrombus formation. With apical ballooning without increased troponin admission levels, there is no indication for OAC. Additionally, patients with midventricular or basal ballooning and normal troponin levels do not require prophylactic OAC [28].

## 2. Chronic Treatment in TTS

**Figure 2:**
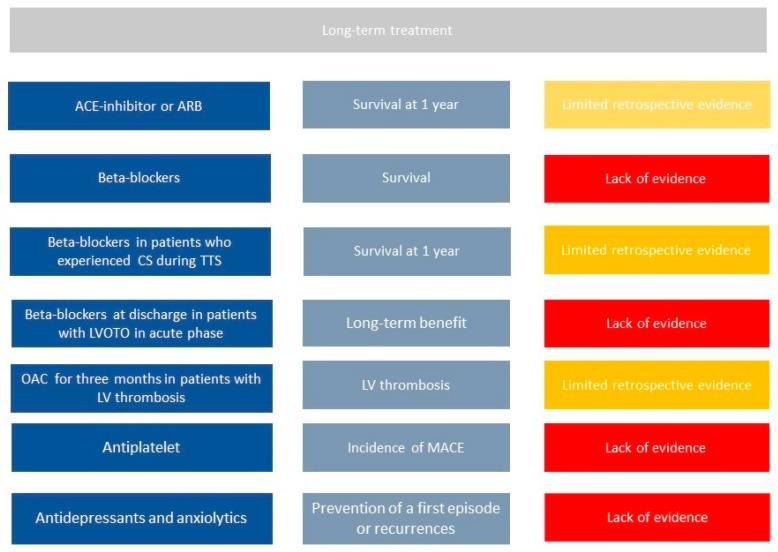
Current literature on long-term management of Takotsubo syndrome patients. First column shows all potential drugs in TTS patients, second one potential benefits, third one current evidence in literature. Legend: CS = cardiogenic shock, LV = left ventricle, LVOTO = left outflow tract obstruction, MACE= mayor cardiac events, OAC = oral anticoagulation, TTS = takotsubo syndrome.

Despite data on long-term outcome from several registries, no randomized trial on pharmacological treatment has been published so far. Therefore, limited information is available and is mainly based on observational studies (Figure 2).

Templin et al., in one of the largest registries on TTS patients, found that the use of angiotensin-converting–enzyme inhibitors (ACEi) or angiotensin-receptor blockers (ARBs) is associated with an improved survival at 1 year; in contrast, according to the same registry, there is no evidence of any survival benefit from the use of beta-blockers (BBs) [3].

Chronic use of BBs has been commonly used on the base that BBs could prevent recurrence of TTs as countering the effect of increased catecholamine levels. However, no benefit in term of recurrence reduction or better survival have been proven [7]. Recently, results from an observational registry (RETAKO) showed that patients with cardiogenic shock who were discharged on BBs experienced lower 1-year all-cause mortality compared with those who did not receive BBs [1].

Long term use of antiplatelet drug still requires investigation. Recent studies showed that aspirin at hospital discharge did not relate to short- or long-term prognosis in a large population of TTS patients. The incidence of MACE in TTS patients discharged with aspirin was not significantly different when compared to patients without aspirin [31]. The protective effect of aspirin in acute cardiovascular diseases, however, is mainly related to the reduction of thrombotic events induced by platelet activation following plaque erosion or rupture. These mechanisms do not seem to play a significant role in TTS [32], because TTS apparently involves coronary microcirculation, and this could explain the lack of potential benefit associated with aspirin in TTS [33]. Therefore, according to current data, the routine use of aspirin should be avoided in TTS patients unless there are clinical conditions with high atherosclerotic risk or history of coronary artery disease as recommended by guidelines.

Oral anticoagulation should be prescribed long term among those patients with evidence of LV thrombi during hospitalization. Indeed, LV thrombi can be successfully managed with 3 months of OAC therapy with complete resolution of thrombosis [28]. Observational data suggest that in selected cases of apical ballooning pattern with increased levels of troponin, prophylactic oral anticoagulation for three months may also be considered.

Recurrence of TTS is not infrequent; the recurrence rate is approximately 1.5% per year [34]. Secondary prevention strategies to avoid such recurrence would be required. However, there is currently no therapy at discharge that proved to reduce the incidence of recurrence at follow-up [6]. Interestingly, a meta regression study that evaluated all published studies found that rates of recurrence are lower in populations treated with combination of ACEi/ARB and BBs [35,36]; however, these results need further investigations.

According to current data, other pathophysiological pathways such as inflammatory or energetic–metabolic need to be further explored to identify a suitable therapy for TTS. A first-line approach remains the management of stressors and comorbidities that can trigger the onset of TTS. Cancer, pulmonary, and neuropsychiatric diseases are the most frequent comorbidities in these patients and should be carefully evaluated during hospitalization and at follow-up. Psychotherapy, counseling, and stress reduction approaches are reasonable and viable options for patients that experienced TTS due to emotional triggers. Therefore, among patients with several comorbidities, especially those with a psychical one, a multidisciplinary long-term approach is needed [37,38].

## 3. Short Term and Long-Term Prognosis

Recent evidence contradicts the assumption that TTS is a benign condition. Indeed, the initial presentation can be accompanied by fatal complications, including cardiogenic shock, congestive heart failure, and lethal arrhythmias, leading to an in-hospital mortality of 2–8.7% [3,4].

### 3.1. Short Term Prognosis

Among patients enrolled in the InterTak registry, during the first 30 days after admission, the rate of major adverse cardiac and cerebrovascular events was 7.1%, which included death and stroke or transient ischemic attack [3].

Cardiogenic shock (CS) during TTS hospitalization is one of the stronger predictors of short and long-term outcome. Patients with CS have 5-fold higher risk of all-cause mortality at long-term. Clinical factors significantly related to CS included male sex, lower LVEF at presentation, longer QTc intervals, the presence of an LVOT gradient, or a physical trigger [1]. Additional risk factors as age > 70 years, history atrial fibrillation, persistent ST elevation during first 72 h of hospitalization, increased levels of NTproBNP at admission and diabetes mellitus were also associated with increased short and long-term mortality risk [39,40,41,42]. Moreover, echocardiography performed during hospitalization has a key role for prediction of cardiogenic shock and death. Indeed, reversible moderate to severe mitral regurgitation and right ventricle involvement are independent predictors of major cardiac adverse events during hospitalization [43,44].

More strict surveillance during follow-up is mandatory for TTS patients with these risk factors.

### 3.2. Long Term Prognosis

At long-term follow-up, the rate of death from any cause was 5.6% per patient-year and rate of major adverse cardiac and cerebrovascular events was 9.9% per patient-year [3].

In an observational study by Stiermaier et al., TTS patients, when compared to ST elevation (STEMI) patients matched for age and gender, presented longer in-hospital stay and significantly higher all-cause mortality during the follow up period (principally related to non-cardiovascular causes). Even the short-term mortality in patients presenting with TTS was higher than generally expected and comparable to a matched STEMI population (mortality rate of 5.5% in TTS patients). This study, moreover, highlighted that male sex, Killip class and diabetes mellitus were predictive factors of long-term mortality, allowing the definition of a prognostic risk score with a point for the presence of each factor. Mortality rates in patients with risk scores from 0 to 3 points were 13.7%, 37.3%, 64.7%, and 100%, respectively [45].

One of the major predictors of long-term prognosis is represented by triggering stressor. Ghadri et al. proposed a patient’s classification according to the type of trigger to predict and stratify short- and long-term outcomes. Patients were stratified into three classes: Class I = triggered by emotional stress, Class II: triggered by physical stress (Class IIa: secondary to physical activities, medical conditions, or procedures, Class IIb: secondary to neurologic disorders), Class III = no identifiable triggering factor. TTS related to neurologic disorders had the highest mortality, while TTS patients with an emotional stressor showed a favorable short- and long-term outcome compared with those with a physical stressor [46]. Uribarri et al. found that TTS triggered by physical factors showed higher mortality at short and long term, and within this group, patients whose physical trigger was hypoxia were those who had a worse prognosis [47].

The incidence of TTS recurrence is estimated to range from 4.7 to 5% at long term follow-up [34,48]. Most recurrences were documented in the first 5 years after the index TTS episode and most baseline characteristics, except for arterial hypertension, were similar between recurrence and nonrecurrence groups of patients. In up to 20% of recurrence cases, a variable TTS pattern is presented. In addition, 46% of patients had a new stress trigger at TTS recurrence. Management and prognostic implication of TTS recurrence require additional investigation.

## 4. Conclusions

Prognosis of patients with TTS is featured by a high risk of major adverse cardiac and non-cardiac events during short and long-term follow-up, due to mainly non-cardiovascular comorbidities. Therefore, careful short- and long-term management is needed in these patients. During the acute phase, TTS is mainly managed with supportive therapy, while, in the chronic phase, a tailored approach based on the cardiovascular and non-cardiovascular risk factors is needed. However, randomized trials are warranted to define the optimal therapeutic approach for TTS patients.
